# Bioethanol Production from Vineyard Waste by Autohydrolysis Pretreatment and Chlorite Delignification via Simultaneous Saccharification and Fermentation

**DOI:** 10.3390/molecules25112606

**Published:** 2020-06-03

**Authors:** Lacrimioara Senila, Eniko Kovacs, Daniela Alexandra Scurtu, Oana Cadar, Anca Becze, Marin Senila, Erika Andrea Levei, Diana Elena Dumitras, Ioan Tenu, Cecilia Roman

**Affiliations:** 1 National Institute for Research and Development of Optoelectronics Bucharest INOE 2000, Research Institute for Analytical Instrumentation subsidiary, 67 Donath Street, 400293 Cluj-Napoca, Romania; eniko.kovacs@icia.ro (E.K.); daniela.scurtu@icia.ro (D.A.S.); oana.cadar@icia.ro (O.C.); anca.becze@icia.ro (A.B.); marin.senila@icia.ro (M.S.); erika.levei@icia.ro (E.A.L.); cici_roman@yahoo.com (C.R.); 2 Faculty of Horticulture, University of Agricultural Sciences and Veterinary Medicine, 3-5 Manastur Street, 400372 Cluj-Napoca, Romania; ddumitras@usamvcluj.ro; 3 Faculty of Agriculture, University of Agricultural Sciences and Veterinary Medicine, 3 Mihail Sadoveanu Alley, 700490 Iasi, Romania; ioantenu@yahoo.com

**Keywords:** vineyard wastes, autohydrolysis method, chlorite delignification, SSF process, bioethanol

## Abstract

In this paper, the production of a second-generation bioethanol from lignocellulosic vineyard cutting wastes was investigated in order to define the optimal operating conditions of the autohydrolysis pretreatment, chlorite delignification and simultaneous saccharification and fermentation (SSF). The autohydrolysis of vine-shoot wastes resulted in liquors containing mainly a mixture of monosaccharides, degradation products and spent solids (rich in cellulose and lignin), with potential utility in obtaining valuable chemicals and bioethanol. The autohydrolysis of the vine-shoot wastes was carried out at 165 and 180 °C for 10 min residence time, and the resulted solid and liquid phases composition were analysed. The resulted liquid fraction contained hemicellulosic sugars as a mixture of alpha (α) and beta (β) sugar anomers, and secondary by-products. The solid fraction was delignified using the sodium chlorite method for the separation of lignin and easier access of enzymes to the cellulosic sugars, and then, converted to ethanol by the SSF process. The maximum bioethanol production (6%) was obtained by autohydrolysis (165 °C), chlorite delignification and SSF process at 37 °C, 10% solid loading, 72 h. The principal component analysis was used to identify the main parameters that influence the chemical compositions of vine-shoot waste for different varieties.

## 1. Introduction

The conversion of the high amounts of wastes generated in vineyards into biofuels is a potential answer to the European Union’s target to find new renewable resources and to reduce the greenhouse emissions [1]. In Romania, the wine production is one of the most important agricultural activities, the wine brands produced in vineyards from Moldova (Iasi, Cotnari, Panciu, Odobesti), Dobrogea (Murfatlar) and Muntenia (Valea Calugareasca) regions being famous all over the world [2]. The by-products generated during the winemaking process are grape stalks, grape pomace, grape seed, wine lees and vine-shoot waste [3,4,5]. Vine-shoots are the main by-product of vineyards, approximately 2–4 tons per hectare being produced from the annual prunings [6].

The most common residues used for the bioethanol production are wood species like eucalyptus, poplar, pine, olive tree, etc. [7]. Bioethanol can be produced also from residues such as vine-shoots with structure comparable to conventional wood and contain lignocellulosic materials with cellulose, lignin and hemicellulose as main components. Cellulose is a polymer containing glucose as monomer, while hemicellulose is a heteropolymer including pentoses (xylose, arabinose), hexoses (glucose, galactose, mannose) and uronic acids [8]. Considering the complex structure of the vine-shoots, its conversion to bioethanol comprises the following steps: (i) separation of carbohydrates from unhydrolyzable components to make them more accessible to the enzymes and reagents, (ii) depolymerization of the carbohydrates into free sugars and (iii) conversion of sugars into ethanol by fermentation. Of these, the carbohydrates separation is the most complex and important step of the process.

Numerous physical, chemical and combined physical-chemical pretreatments are used for the conversion of biomass into sugars, before fermentation [9,10]. Physical pretreatments are used for size reduction and materials’ porosity increase and include: mechanical comminution, extrusion and irradiation. Chemical pretreatments with acid or alkaline reagents are used to destroy the biomass structure. The combination of physical and chemical pretreatment improves the conversion yields. The commonly used pretreatments for lignocellulosic separation include: steam explosion, dilute acid, ammonia explosion and autohydrolysis. Each pretreatment has advantages and disadvantages and often have a selective action. Autohydrolysis is an environmentally friendly pretreatment method using only water as reaction medium, at high temperature (170–230 °C) and pressure (up to 5 MPa) [11,12,13].

The vine-shoot wastes are used in the production of cosmetics, biopolymers and coatings [14], lignin and cellulose [15], chemicals (like lactic acid, furfural), heat and electricity [16], lignin and glucose [17], and biomolecules [18]. Currently, the C_6_-sugars derived from lignocellulosic biomass receive more attention than the C_5_-sugars obtained from hemicelluloses. Xining et al. [19] analysed the monosaccharides (glucose and xylose), xylo-oligosaccharide, furfural and 5-hydroxy-methylfurfural (HMF) in the liquid fraction resulted after the autohydrolysis of wheat straw. Cebreiros et al. [20] investigated the autohydrolysis and organosolv delignification of eucalyptus sawdust and the chemical compositions of the pretreatment liquid.

Lignin contains *p*-coumaryl alcohol (H-lignin), coniferyl alcohol (G-lignin), and sinapyl alcohol (S-lignin) in variable proportions, depending on the biomass type [12]. Delignification is the process of lignin removal for increasing the accessibility of enzymes to the fermentable sugars. Various lignin removal methods were reported: alkali pretreatment, kraft pulping, acid hydrolysis, ionic liquid and acidified sodium chlorite [21]. The alkali treatment dissolves lignin, but has the disadvantage of decreasing the amorphous cellulose and consequently decreases the enzymatic hydrolysis yield. The acid hydrolysis affects very slowly the lignin structure, while the ionic liquids selectively bond the lignin and change the structure of cellulose [21]. The kraft pulping is the industrial scale method used for the lignin removal from wood, but it also degrades the cellulosic sugars [22]. Among all methods, the acid-chlorite delignification is the most selective because the chlorite reacts only with phenylpropane units from the lignin structure [22]. The disadvantage of this delignification method is the necessity to use the appropriate reagent for a selective reaction. The production of carbohydrates from vineyard vine-shoot waste (Feteasca Regala variety) by microwave pretreatment, acid-chlorite delignification and enzymatic hydrolysis was reported, with a yield of 14.0 g of glucose from 100 g biomass [23]. The processes used for the fermentation to bioethanol are: separate hydrolysis and fermentation (SHF), simultaneous saccharification and fermentation (SSF), batch fermentation, feed batch fermentation and solid-state fermentation [8]. The SSF process has many advantages, compared to the SHF: hydrolysis and fermentation occur simultaneously in the same reactor where enzymes and yeast are put together and glucose is immediately converted into ethanol. The main drawback of this method is the difficulty to find the optimal temperature for hydrolysis and fermentation. Generally, *S. cerevisiae* has the best performance at 30 °C, while the cellulolytic enzymes are active around 45–50 °C [24,25]. Generally, a high quantity of fertilizers and copper-based substances are used for vineyard growth. The interference between soil and vine-shoots produces the adsorption of metals and their binding to the organic part [26,27].

The autohydrolysis of vine-shoot waste varieties performed at 150 °C revealed that *Vitis vinifera* cultivars are potential source for producing valuable compounds, among them also bioethanol [28]. In this regard, this study aims to continue the investigation and to compare bioethanol production by autohydrolysis pretreatment, acid chlorite delignification, followed by simultaneous saccharification and fermentation from vineyard waste resulted from several varieties. Also, the mineral, protein, elemental analysis and calorific value of vine-shoot wastes were investigated in correlation with their polymeric compositions (cellulose, hemicelluloses, and lignin content).

## 2. Results and Discussion

### 2.1. Chemical Composition of Vine-Shoot Waste

The chemical composition of the studied eight vine-shoot wastes resulted from *Vitis vinifera* cultivars was previously reported [28]. The cellulose, hemicellulose and lignin contents were in the range of 28.8–40.4%, 17.3–28.0%, and 25.9–32.6%, respectively. The high content of holocellulose (mixture between cellulose and hemicellulose) ranging between 51.3% (Muscat Ottonel variety) and 64.6% (Cabernet Sauvignon variety) recommends the vine-shoots as raw material for the bioethanol production. The highest content of cellulose (40.4%) was found in the Pinot Noir variety.

### 2.2. Autohydrolysis Pretreatment of Vine-Shoot Wastes

By pretreatment, the vine-shoot matrix is destroyed and the enzymes’ access to the carbohydrates is facilitated. Based on our previous studies, the autohydrolysis was performed at 150 °C for 10 min, the reported solid yield ranging between 60.5–75.0% [28,29]. In this study, the dependence of solid yield on the pretreatment operating temperature and the vines-shoot waste variety was observed. In Table 1 is presented the chemical compositions of vine-shoot waste and solid yield and chemical compositions of autohydrolysed vine-shoot waste obtained at 165 °C and 180 °C. The solid yields for the used pretreatments were found to be between 62.2 and 71.0% (165 °C) and 52.3 and 64.3% (180 °C), respectively. Jesus et al. [30] also reported the dependence of the yield on the operating temperature of the autohydrolysis of vine pruning residues, obtaining a solid yield of 86.4–62.4% and 66.0–63.6% raw material by autohydrolysis at 180 °C (for 10 to 80 min) and 200 °C (for 30 to 90 min), respectively. In our experiments, the resulted yields were lower compared to those reported by Jesus et al. due to the high operating pressure (60 bars) [30].

The cellulose content of the autohydrolysed materials also increased with increasing autohydrolysis temperature from 38.3–47.4% at 150 °C in our previous study [28], to 49.3–57.3% at 165 °C and then slightly decreased to 45.3–53.3% at 180 °C in the present study. Thus, the optimal pretreatment temperature for obtaining high cellulose content in pretreated vine-shoot wastes was found to be 165 °C.

The lignin content of the autohydrolysed material varied as follows: 22.4–35.2% (150 °C) [28], 32.4–45.0% (165 °C) and 28.4–41.0% (180 °C). The hemicellulose content of the pretreated solids decreased with the pretreatment; at 180 °C the hemicellulose being totally solubilized in the liquid fraction. A small quantity of hemicellulose was found in the solid fraction, which led to an increased content of cellulose and lignin in the pretreated samples, due to the concentration effect [31].

#### Chemical Compositions of Liquid Samples

**Figure 1:**
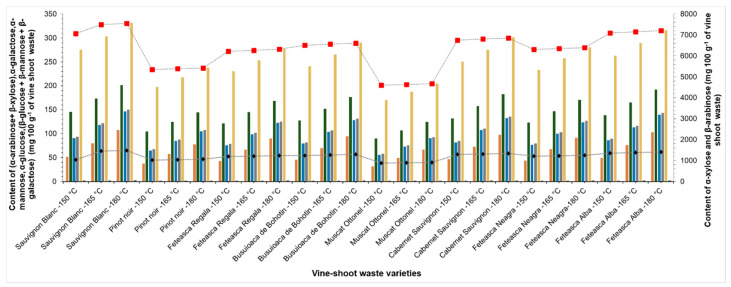
Content of anomers from vine-shoots waste hemicellulosic sugars as functions of the vine plant varieties (α-arabinose + β-xylose, α-galactose, α-mannose, α-glucose, a β-glucose + β-mannose + β-galactose, β-arabinose and α-xylose).

In recent years, various methods were applied for sugars analysis in liquid samples, but the information regarding each sugar isomer in the liquid fraction resulted after the vine-shoot waste autohydrolysis is not available [32]. The mono-sugars content of liquid samples resulted from the autohydrolysis was analysed by gas chromatography coupled with mass spectrometry (GC-MS) after derivatization. By derivatization, the large molecules of hemicellulosic sugars from the liquid fraction were transformed in more volatile compounds that can be analysed by GC. The derivatization of mono-sugars from the hemicellulosic fraction was performed in two steps, namely oximation and silylation, with *N*,*O*-bis(trimethylsilyl)trifluoro-acetamide (BSTFA), providing an efficient conversion of the monosugars into a mixture of two anomers (α and β) [32,33]. According to Herde et al. [34], the long reaction time of hemicellulosic sugars led to a lower yield of derivatization. The results show that some isomers overlap due to their close values of the retention time, therefore they will be quantified together, which is the case of α-arabinose + β-xylose and β-glucose + β-mannose + β-galactose. Figure 1 presents the content of anomers obtained from hemicellulosic sugars separated from different vine plant varieties. The results suggested that the highest amount was found for the β-arabinose anomer (between 4.3 and 7.5% of vine-shoot waste), followed by the α-xylose (between 0.9 and 1.4% of vine-shoot waste). According to the obtained results, the pretreatment temperature increase led to an increased content of the mono-sugars identified in the liquid fraction.

Our results indicated that, in all samples, β-arabinose is the major sugar. Prozil et al. [35] reported that monosaccharides from grape stalks of *Vitis vinifera* L. contain: xylose (20.4%), glucose (62.7%), arabinose (5.5%), mannose (4.8%) and galactose (4.9%). The results proved that vine-shoot waste hemicellulose contains aldopentoses with a high content of arabinose and xylose.

**Figure 2:**
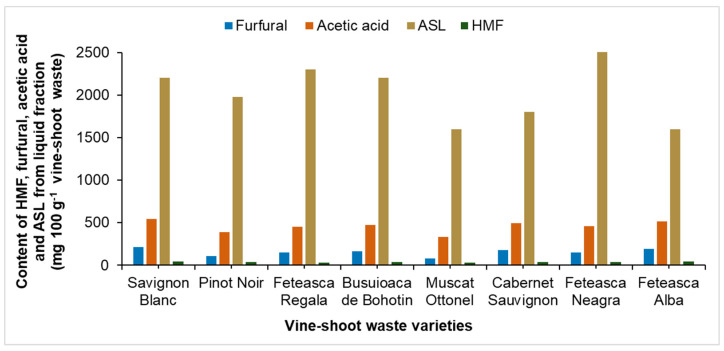
The content of HMF, furfural, acetic acid and ASL in the hemicellulosic fraction separated after autohydrolysis at 180 °C.

Figure 2 presents the content of HMF, furfural, acetic acid and acid-soluble lignin (ASL) obtained from a pretreatment performed at 180 °C. The content of HMF was between 32.1 and 40.5 mg 100 g^−1^ vine-shoot waste and the furfural content ranged between 81.0 and 212 mg 100 g^−1^ vine-shoot waste.

**Figure 3:**
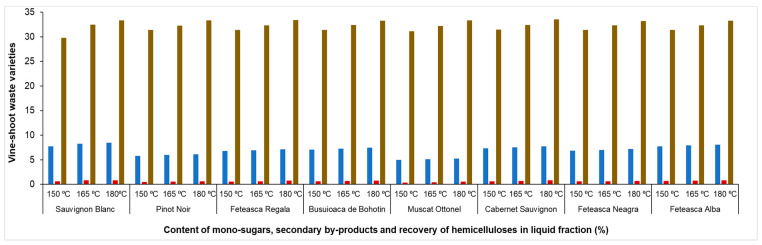
Recovery of hemicellulose in the liquid fraction for autohydrolysis performed at each temperature (mono-sugars, secondary by-products and hemicellulose).

Recovery of hemicellulose in the liquid fraction resulted after autohydrolysis pretreatment is shown in Figure 3. The hemicellulose recovery in the liquid fraction was between 29.7% (Sauvignon Blanc variety for pretreatment performed at 150 °C) and 33.5% (Cabernet Sauvignon variety for pretreatment performed at 180 °C). The hemicellulose was recovered in the liquid fraction as mono-sugars and secondary by-products (furfural, HMF and acetic acid). The recovery of hemicellulose in liquid samples was calculated taking into account the sum of mono-sugars and secondary by-products. The highest recovery percent of hemicellulose was obtained for experiments performed at 180 °C due to the increasing of by-products concentration with the temperature.

The mono-sugars obtained in the liquid fraction, resulting from the biorefinery process, can be a subject of further research for valorisation, as platforms for the synthesis of other chemicals.

### 2.3. Delignification of Pretreated Vine-Shoot Waste

**Figure 4:**
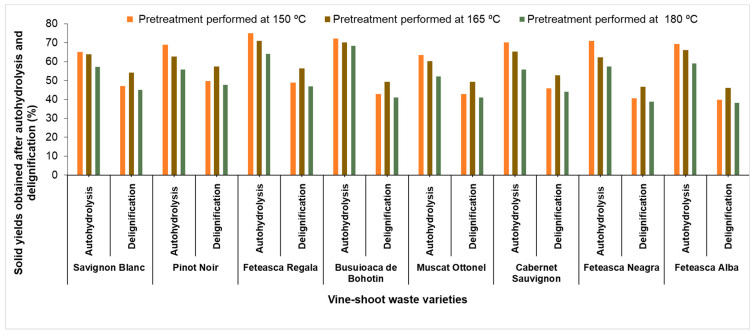
Solid yields obtained after delignification and pretreatment of shoot wastes of the studied vine-shoot varieties.

According to Sánchez-Gómez et al. [36], the vine-shoot waste contains high concentrations of tannins, which are structurally associated with lignin and make the separation of lignin difficult, due to the formation of alkali-aryl condensed structures. By applying a delignification method with sodium chlorite to the autohydrolysed vine-shoot waste, lignin was oxidized, as indicated by the colour changing from brown to yellow. The solid yields of the delignified vine-shoot waste and the solid yields of the pretreated vine-shoot waste are presented in Figure 4.

**Figure 5:**
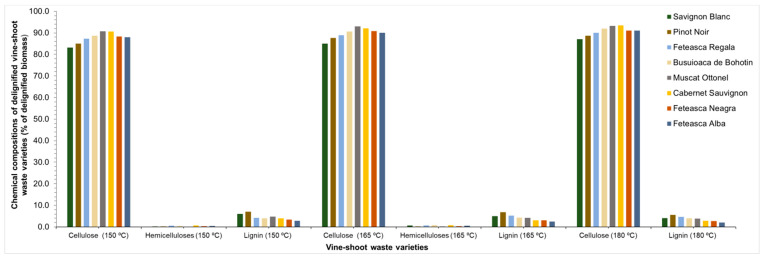
Chemical compositions of residual solids after delignification of pretreated shoot wastes of the studied vine plant varieties.

The solid yield after delignification of the vine-shoot waste varied in the range of 40.6 to 49.8% for a pretreatment performed at 150 °C, in the range of 46.0 to 57.4% for a pretreatment performed at 165 °C and between 38.4 and 47.2% for a pretreatment carried out at 180 °C (Figure 4). The solid yields of delignified material depend on the vine-shoot waste variety used and on the temperature. The content of cellulose and trace of hemicellulose and lignin of the delignified substrate obtained for each variety was analysed. The content of cellulose increased significantly after the delignification in all varieties and was between 83.0 and 90.7%, 85.0 and 93.0%, and 87.0 and 93.5% for a pretreatment performed at 150 °C, 165 °C and 180 °C, respectively. The low lignin content found confirms its almost complete elimination after the delignification stage. The hemicellulose content was in the range 0.2–0.8%, whereas for a pretreatment performed at 180 °C, the hemicelluloses were totally eliminated (Figure 5).

#### Mass Balance after Pretreatment and Delignification

The balance of materials was calculated after the pretreatment and delignification processes in order to determine the cellulose and lignin content.

The results presented in Table 2 showed that, after acid chlorite delignification, the cellulose represents 18.0–25.0% of biomass. The highest content of cellulose was obtained for vine-shoot waste pretreated at 165 °C and varied depending on the treated variety (ranging from 20.0 to 24.7% of vine-shoot waste).

### 2.4. Effect of Pretreatment and Delignification on the Vine-Shoot Waste Structure

#### 2.4.1. SEM Analysis

**Figure 6:**
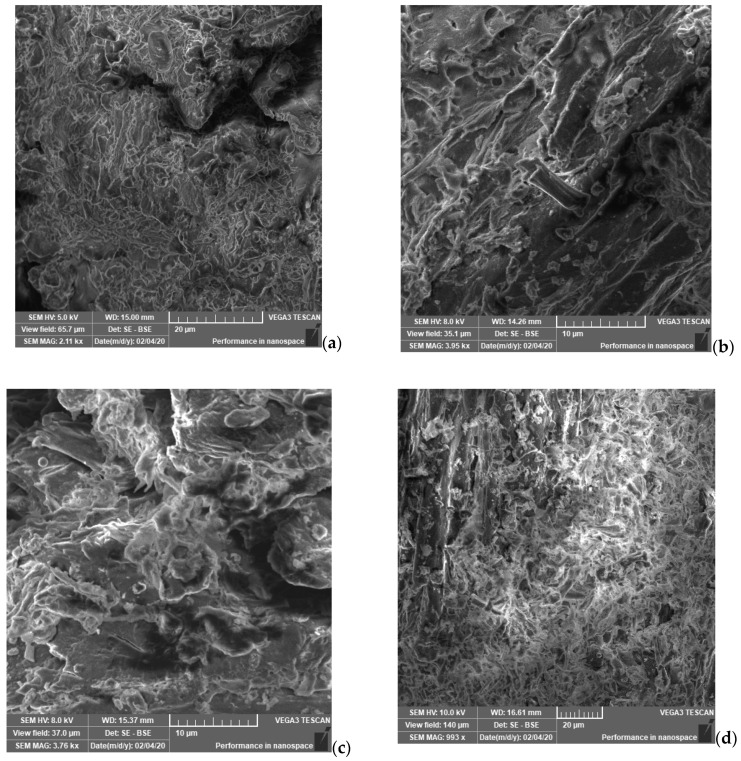

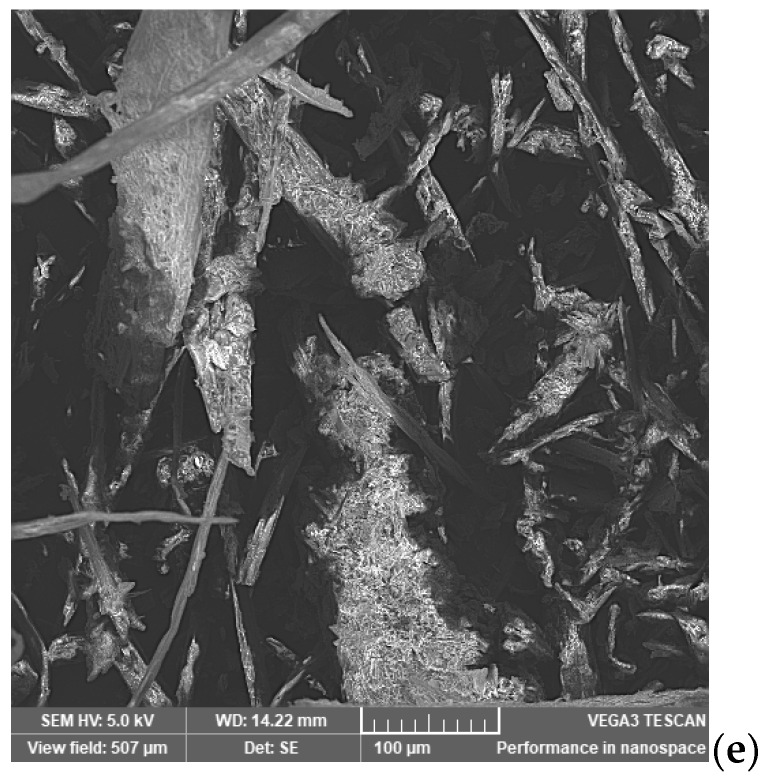
Scanning electron microscopy images of the samples (vine-shoot waste of Busuioaca de Bohotin variety): (**a**) untreated, (**b**) autohydrolysed at 150 °C, (**c**) autohydrolysed at 165 °C, (**d**) autohydrolysed at 180 °C, (**e**) delignified.

The microstructure of some autohydrolysed and delignified vine-shoots can be seen in SEM images (Figure 6). The vine-shoots structure was unaltered, but after the pretreatment it became amorphous and porous, while after delignification it was completely destroyed, with open chains. The pretreatment temperature changes the microstructure of vine-shoot waste and by increasing the temperature a more porous, defragmented and open structure was observed. The effect of the pretreatment sequence and the resulting destroyed structure of the vine- shoot waste, reported in various studies, converge with our results [30,37].

#### 2.4.2. XRD Analysis

**Figure 7:**
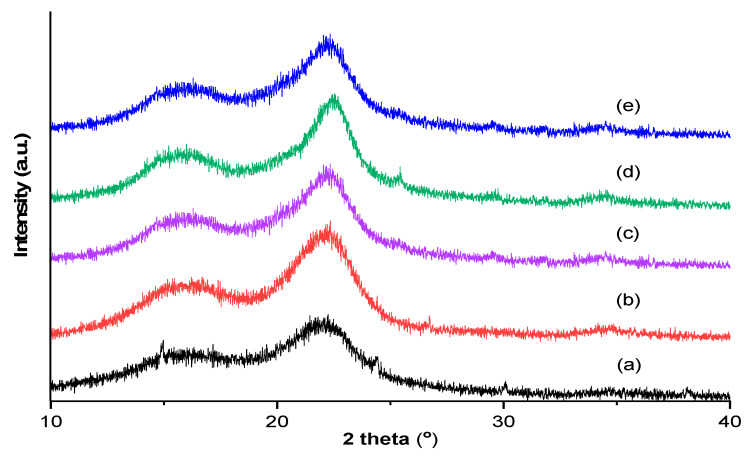
XRD patterns of the vine-shoot waste (Busuioaca de Bohotin variety) samples: (**a**) untreated, (**b**) delignified, (**c**) autohydrolysed at 150 °C, (**d**) autohydrolysed at 165 °C and (**e**) autohydrolysed at 180 °C.

The XRD spectra of untreated, autohydrolysed and delignified samples (Busuioaca de Bohotin variety) were presented in Figure 7. The crystallinity index (CrI), degree of crystallinity (χ_c_) and crystallite size (Dc) were calculated in order to evaluate the crystallinity behaviour of the samples. The obtained results are presented in Table 3.

The X-ray patterns of all vine-shoot waste samples showed the same diffraction features as crystalline cellulose. The presence of both crystalline and amorphous cellulose is indicated by the two major peaks (2θ around 18° and 22.5°). The intensities of these two peaks indicated the predominance of cellulose I structure in all samples. The presence of hemicellulose and lignin was not indicated by the XRD pattern. Similar results were obtained for the argan wood [38]. Achaby et al. [39] identified three major peaks at 2θ at 15.5°, 16.4° and 22.8° for the pure cellulose nanocrystals obtained from vine-shoot waste. In our case, the pretreated vine-shoots contain, besides cellulose, also traces of hemicellulose and lignin.

The crystallinity of cellulose during the process of pretreatment and delignification can be affected. The CrI decreases from 78.6% (untreated vine-shoot waste) to 61.0–71.3% (pretreated vine-shoot waste) demonstrating that autohydrolysis pretreatment is suitable for obtaining amorphous cellulose ready for enzymatic hydrolysis. A possible explanation could be the hemicellulose removal and the increase of the amorphous cellulose content. Davila et al. [15] reported that the substrate resulted after the microwave alkali pretreatment has a high crystallinity index, due to the elimination of amorphous cellulose after pretreatment. According to Xu et al. [40], the CrI is inversely correlated with the enzymatic hydrolysis rate.

### 2.5. Simultaneous Saccharification and Fermentation Process

**Figure 8:**
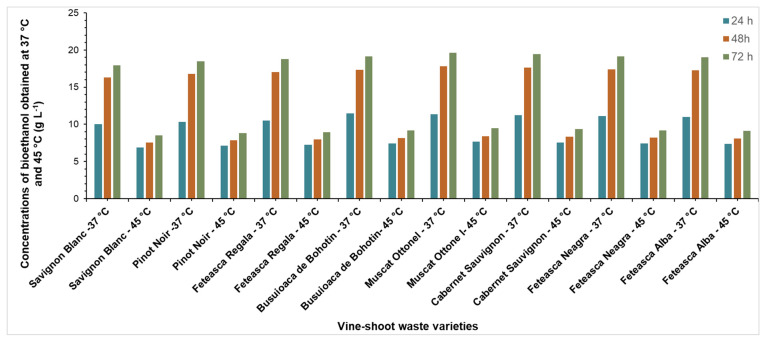
Bioethanol concentration obtained by SSF of vine-shoot waste at 37 °C and 45 °C for pretreatment performed at 165 °C.

The pretreatment by autohydrolysis and delignification with acid chlorite effectively destroyed the vine-shoot waste, by eliminating the hemicelluloses and then the lignin. In this study, the substrates obtained from all the varieties, after delignification, were subjected to saccharification and fermentation to obtain bioethanol. All SSF experiments were performed by using substrate loadings 10% and two temperatures (37 °C and 45 °C). In the SSF process, glucose was simultaneously produced and consumed [30]. A complex enzyme cellulase from *Trichoderma reesei*, the ATCC 26921, and β-glycosidase were used to decompose cellulose into monomeric sugars which are converted into bioethanol. In order to increase the bioethanol yield, two temperatures and reaction times were tested. The bioethanol concentration obtained by the SSF process from vine-shoot samples, at 37 °C and 45 °C is presented in Figure 8.

The highest concentrations of ethanol—between 17.9 to 19.6 g L^−1^—were obtained at 37 °C for each vine-shoot waste variety, whereas at 45 °C, the ethanol concentrations were between 8.5 to 9.3 g L^−1^. The ethanol amounts resulting from the SSF process are strongly influenced by the reaction temperature. This could be attributable to the reactivity and the complex cellulase enzymes used. The complex enzyme used in the SSF process, cellulase from *Trichoderma reesei* the ATCC 26921, has a non-porous nano silica and the efficiency is influenced by the temperature. The SSF process combines both the enzymatic hydrolysis (where the temperature for enzyme activation is between 40 to 60 °C) and the fermentation (the temperature for an optimal *S. cerevisiae* growth rate is around 30 °C). The temperatures chosen for this study were selected to cover reactivity domain, for both enzymes and yeast, and to study the dependence of enzymes efficiency on temperature.

The low concentration of bioethanol obtained at 45 °C proved that high temperatures decrease the yeast productivity. The optimal temperature for the vine-shoots waste SSF process was at 37 °C, probably due to the yeast physiology. The *S. cerevisiae* yeast was not suitable for high temperature fermentation due to induction of stress and probable morphological changes in yeast during fermentation [41]. These results suggest that for this strain, the optimum SSF temperature should be below 45 °C under the conditions used in this study.

**Figure 9:**
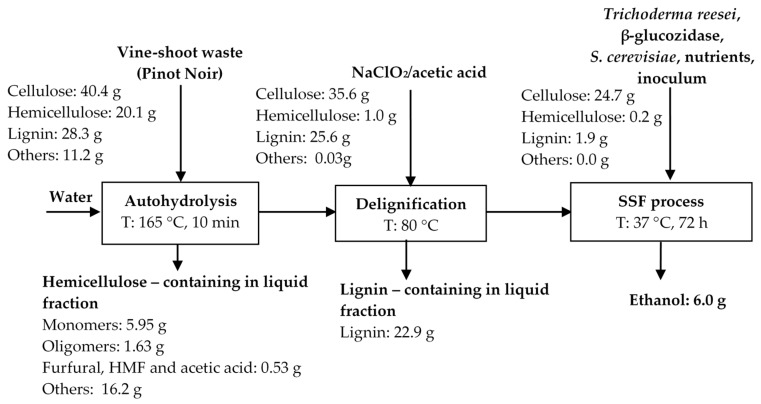
Material balance for bioethanol production from Pinot Noir variety of vine-shoot waste.

The material balance for bioethanol production from the Pinot Noir variety is presented in Figure 9.

In Table 4 ethanol concentrations obtained in the current study are compared with those reported in literature for a large variety of lignocellulosic biomass.

### 2.6. Physico-Chemical Characteristics of Vine-Shoot Waste Variety

The content of minerals and calorific value from the studied vine-shoot waste varieties is presented in Table 5.

High amounts of K, Ca, Mg and Na were found in all vine-shoot wastes and their content varied in the range (mg kg^−1^): 1788–3414 (K), 1622–2862 (Ca), 719–1255 (Mg) and 16–364 (Na). The content of Al ranged between 41.7 and 67.0 mg kg^−1^. In our previous study [28], the content of some trace metals, were reported to be in the ranges of 30.0–80.3 mg kg^−1^ (Fe), 10.4–16.1 mg kg^−1^ (Cu), 6.1–15.8 mg kg^−1^ (Zn), 10.7−18.0 mg kg^−1^ (Mn), 18.7–35.5 mg kg^−1^ (Sr) and 14.7–28.8 mg kg^−1^ (Ba). Also, content of protein, C and H were reported to be in the range of 5.2–10.0% protein, 43.1–44.6% C and 5.83–6.23% H. The high calorific value of all vine-shoot wastes analysed in this study appears to be an attractive option for the production of valuable compounds, such as pellets and briquettes.

### 2.7. Results Characterization Using Principal Component Analysis

In order to find the possible influence of the chemical composition of vine-shoot wastes on the bioethanol production principal component analysis (PCA) was applied. Four principal components (PC) explain about 86% of the data variability. The varimax rotated factor loadings of the first 4 PC’s are presented in Table 6.

**Figure 10:**
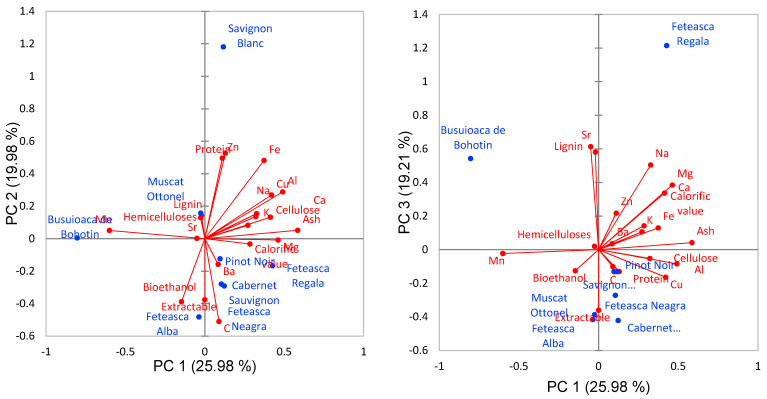
PCA biplots.

The loadings in boldface correspond to the variables with a dominant influence on the selected PC. PC1 explained 39.8% of the variance, having a positive loading on ash, cellulose, Cu, Mg, Ca and Al and a negative loading on Mn. According to Zając et al. [45], biomass ash contains a high amount of Cu and other elements such as Ca, Mg, K, Na, P, S and Zn. Also, Ullah et al. [46] reported that heavy metals in the rice plant showed a positive correlation with each other (Cd, As, Pb, Cr) and negative correlation with Fe and Mn. PC2 explained 18.8% of the system variability and showed a positive loading on protein, Fe and Zn and a negative loading on extractable, C and bioethanol, as these parameters play an important element for plant growing and act as a cofactor for protein. PC3 explained 14.7% of the variability and showed positive loadings on lignin, Na, Mg, Ca and Sr and a negative correlation with extractable. PC4 explained about 12% of the total variance of the system and revealed a positive correlation on hemicellulose, K and Ba. Similar results were reported by Kim et al. [47] who studied the effect of non-structural organics and inorganics constituents of switchgrass during pyrolysis and found a positive correlation between the removal of hemicelluloses and K. A possible explanation is that K has a strong catalytic interaction with carbohydrates. In Figure 10 is presented the PCA biplots.

**Figure 11:**
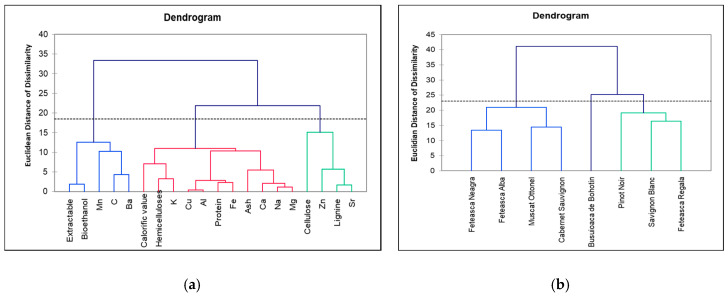
Hierarchical clustering (dendrogram) of (**a**) different components of vine-shoot waste (**b**) vine-shoot waste varieties.

The PC1-PC2 biplot shows that Pinot Noir, Feteasca Regala, Cabernet Sauvignon and Feteasca Neagra contain higher levels of mineral content, calorific value, cellulose and ash than Busuioaca de Bohotin, Muscat Ottonel and Feteasca Alba. On the other hand, Sauvignon Blanc contains higher Zn and protein content than the other varieties High levels of Mn but low levels of other elements contain the wastes of Busuioaca de Bohotin variety. Similar calorific value, C, Mg and Ba contents presents the Pinot Noir, Feteasca Regala, Cabernet Sauvignon and Feteasca Neagra due to calorific value, C, Mg and Ba content. The Feteasca Alba shoot waste differentiate by the bioethanol and extractable contents while Sauvignon Blanc due to protein, Zn, Fe, Cu Al, Na, Ca, K, cellulose and ash content. A good degree of discrimination of the vine plant variety was noticed according to their chemical compositions. The similarity between different parameters of vine-shoot waste was realized by using a hierarchical clustering (dendrogram form) (Figure 11).

Hierarchical clustering analysis (HCA) group the vineyard vine-shoot waste based on a set of variables that represents the main characteristic of the vine-shoot wastes. Garcia et al. [48] used multivariate statistics for an estimation of the potentials of various types of lignocellulosic biomass for the biofuels production. The dendrogram from Figure 11b cluster the vine-shoot wastes into 3 groups, the first containing Feteasca Neagra, Feteasca Alba, Muscat Ottonel and Cabernet Sauvignon characterized by low lignin and high extractable content, the second group containing Pinot Noir, Sauvignon Blanc and Pinot Noir, characterized by high cellulose and calorific value and a third group containing Busuioaca de Bohotin, characterized by high Mn content. The mineral compositions reflect the environment in which the grapevines are cultivated. According to Figure 11a, the cellulose is linked with Zn and Sr. According to Antonkiewicz et al. [49], cellulose correlates with heavy metals content and in particular, the Zn, Cd and Pb that increases with the cellulose content. The metals can be bound by vine-shoot through ion exchange, specific absorption or complexation with organic compounds (like cellulose, hemicellulose or lignin). Extractable are correlated with organic carbon content, since they consist in carbon rich fats.

## 3. Materials and Methods

### 3.1. Chemicals and Reagents

All used chemicals were of analytical reagent grade. Acetic acid, dichloromethane, sulfuric acid (98%), sodium hydroxide, methanol, hydrochloric acid, suprapure nitric acid 65%, ethanol, toluene, acetone, hydrogen peroxide 30%, glucose, mannose, galactose, arabinose, pyridine, hydroxylamine hydrochloride (NH_2_OH·HCl) and salicin were purchased from Merck (Darmstadt, Germany). Sodium chlorite (80%) was purchased from Alfa Aesar GmbH & Co (Karlsruhe, Germany). Enzymes cellulase from *Trichoderma reesei* ATCC 26921, β-glucosidase from almonds, *S. cerevisiae* YSC2, peptone, BSTFA, 5-hydroxymethylfurfural and furfural were purchased from Sigma-Aldrich (St. Louis, MO, USA). *Trichoderma reesei* ATCC 26921 is a lyophilized powder with concentration ≥1.0 unit per mg of solid. All solutions were prepared by using ultrapure water (18.2 MΩcm^−1^ at 20 °C) obtained from a Direct-Q3 UV Water Purification System (Millipore, Molsheim, France).

### 3.2. Sample Description

**Figure 12:**
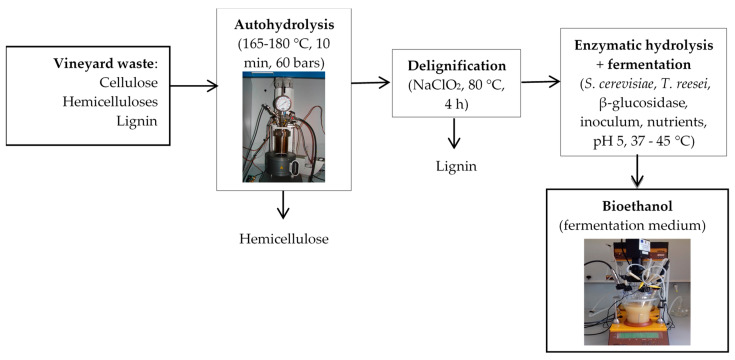
Schematic representation of the bioethanol production from vineyard wastes.

The vine-shoot wastes were procured from the Research Station of the University of Agricultural Sciences “Ion Ionescu de la Brad” from Iasi, farm no. 3 “Vasile Adamachi” (Romania). The vine plant varieties were: Sauvignon Blanc, Pinot Noir, Feteasca Alba, Feteasca Regala, Feteasca Neagra, Busuioaca de Bohotin, Cabernet Sauvignon and Muscat Ottonel. The vine-shoots were collected immediately after the pruning operations in 2019, oven-dried at 60 °C for 24 h, and then cutter-milled to pass through a 2 mm pore size sieve. The samples were stored in plastic bags in the dark, at room temperature, until use. The scheme for bioethanol production from vine-shoot waste is presented in Figure 12.

### 3.3. Autohydrolysis Pretreatment

The vine-shoot waste was pretreated by autohydrolysis, using a steel pressure Parr reactor (Parr Instruments, Moline, IL, USA), equipped with a Parr 4523 temperature controller and a 1 L reaction vessel. The raw material and the water were mixed at 1:7 solid to liquid ratio. The mixture was heated to 165 and 180 °C, for 10 min at each temperature. The preliminary experiments performed for autohydrolysis of vine-shoot waste at 150 °C were reported in our recent work [28]. The obtained previous results were used in this study for comparison and the autohydrolysed product was further processed for bioethanol production. At the end of the reaction, the samples were cooled at room temperature, the solid fraction was separated by filtration and the chemical composition was analysed.

### 3.4. Acid Chlorite Delignification

For the acid chlorite delignification, the vine-shoot wastes were treated with NaClO_2_ in an aqueous solution of 10% acetic acid, according to the Browning method [50]. The experiments were conducted in a 250 mL flask containing NaClO_2_ (0.6 g g^−1^ biomass, dry biomass), acetic acid (0.6 mL g^−1^ biomass, 10%, dry biomass) and water (84 mL g^−1^ biomass, dry biomass). The reactions took place at 80 °C for 2 h, after each reaction, the biomass being washed with water. The process was repeated 3 times. The slurry was filtered, washed with water and acetone to neutral pH. The delignified material was filtered and dried at 105 °C for 24 h and analysed for cellulose, lignin and hemicellulose content. The acid chlorite delignified samples were subjected to SSF method for obtaining bioethanol.

### 3.5. Production of Bioethanol from Delignified Vine-Shoots Waste

#### 3.5.1. Microorganism and Inoculum Preparation

*S. cerevisiae* YSC2 was maintained in Petri dishes containing potato dextrose sugar agar culture medium at 30 °C for 24 h. The inoculum strains were grown in 250 mL Erlenmeyer flasks with orbital shaking (150 rpm) in a sterile culture media containing 50 g L^−1^ glucose, 10 g L^−1^ yeast extract, 20 g L^−1^ peptone, 20 g L^−1^ (NH_4_)_2_SO_4_, 20 g L^−1^ KH_2_PO_4_, 10 g L^−1^ of MgSO_4_·7H_2_O at 30 °C for 12 h. The nutrient solution contained per liter: 5 g yeast extract, 20 g KH_2_PO_4_, 10 g of MgSO_4_·7H_2_O and 20 g (NH_4_)_2_SO_4_.

#### 3.5.2. Simultaneous Saccharification and Fermentation of Processed Vine-Shoots Waste

The SSF experiments on the solid residue, recovered after the delignification of the pretreated vine-shoot wastes, were carried out in a 2 L bioreactor (Lambda Minifor, Lambda Laboratory Instruments, Brno, Czech Republic) equipped with sensors for dissolved oxygen, pH and temperature. SSF media were prepared by mixing nutrient solution (150 mL), inoculum solution (150 mL) with 10% (*w*/*v*) solids loadings in citrate buffer (0.05 M) and enzymes at pH = 5 and reacted at two temperatures (37 and 45 °C) for 72 h. The cellulase from *Trichoderma reesei* ATCC 26921 and β-glucosidase from almonds were used for the SSF experiments. In all cases, 10 FPU g^−1^ substrate of enzymes cellulase from *Trichoderma reesei* ATCC 26921 and 20 Ug^−1^ substrate β-glucosidase from almonds were used. During the SSF process, the samples (1 mL) were taken and centrifuged at 5000 rpm for 10 min and used for ethanol analysis.

### 3.6. Chemical Characterization of Raw, Autohydrolysed and Delignified Materials

#### 3.6.1. Cellulose, Hemicelluloses and Lignin Content for Raw, Autohydrolysed and Delignified Materials

The content of holocellulose (the mixtures of cellulose and hemicelluloses) from raw, autohydrolysed and delignified materials was determined from the remaining residue after the reaction with NaClO_2_. 2.5 g of the sample were refluxed with 2.5 g NaClO_2_ in 10% acetic acid at 75 °C for 1 h (the process was repeated 4 times). The obtained holocellulose product was filtered, dried at 105 °C for 24 h and weighed. The cellulose content was determined as the holocellulose residue insoluble in NaOH (17.5%). 1 g of the previously obtained holocellulose sample was reacted with 25 mL NaOH (17.5%) at 20 °C for 40 min. The resulting residue was washed with water, dried at 105 °C for 48 h and weighed. Hemicellulose content was calculated as the difference between the holocellulose and cellulose content. The lignin content was determined as the insoluble residue in 72% H_2_SO_4_ solution. 1 g of dried biomass was refluxed with 72% H_2_SO_4_ for 4 h at 20 °C. 500 mL of distilled water was added to the resulting solution, and then the final was refluxed another 4 h. The residue was filtered, washed with water, dried at 105 °C for 24 h and weighed. The lignin content (%) was determined as the ratio between the lignin (g) and the initial mass (g) [51].

#### 3.6.2. Metals Determination in Untreated Vine-Shoot Waste Samples

An amount of 1 g of sample was digested with 5 mL nitric acid 65% and 2 mL hydrogen peroxide 30% in closed polytetrafluoroethylene (PTFE) vessels, using a microwave digestion system (Speedwave MWS-3+, Berghof, Eningen, Germany) according to the method described by Senila et al. [52]. The digested samples were quantitatively transferred into 20 mL volumetric flasks and diluted to the mark with ultrapure water. Three replicated measurements were carried out for each sample. The metal contents were measured using an Inductively Coupled Plasma Optical Emission Spectrometer (ICP-OES) Optima 5300 DV (Perkin Elmer, Woodbridge, ON, Canada). The calibration standards were prepared from 1000 mg L^−1^ of multielement standard solutions (Merck) containing all the investigated elements by appropriate dilutions.

#### 3.6.3. Determination of Calorific Value

The gross calorific value was determined by a 6200 Isoperibol calorimeter (Parr Instruments), calibrated by combustion of certified benzoic acid. The dried biomass was analysed as given in the Standard Methods (DIN 51900-1:2000) [53]. The sample was placed in the sampler holder of the bomb. The bomb was assembled, filled with oxygen for 30 s at a pressure of 400 psi and placed in the calorimeter. The sample was burned under controlled conditions for 15 min.

#### 3.6.4. Determination of Hemicellulose Monosaccharides by GC-MS

The liquid fraction resulted after the autohydrolysis pretreatment was separated by filtration and then subjected to carbohydrate analyses by GC-MS according to our previous publication [32]. Before GC-MS analysis, about 20 mL liquid samples were extracted with a mixture of dichloromethane and methanol (2:1 *v*/*v*). The extract was evaporated to dryness and then it was derivatized by using 2.5% hydroxylamine hydrochloride in pyridine at 80 °C for 30 min and then silylated with 300 µL BSTFA at 80 °C for 10 min. The derivatized solutions were injected into the GC-MS.

#### 3.6.5. Determination of HMF, Furfural, ASL and Acetic Acid from Hemicellulosic Fraction

The liquid fraction resulted after the autohydrolysis pretreatment was extracted with 15 mL dichloromethane for HMF determination. The extract was dried under nitrogen and then it was derivatized with 300 µL BSTFA and heated at 60 °C for 10 min and then determined by GC-MS [54]. The furfural content was determined by GC-MS after the extraction of liquid fraction resulted after autohydrolysis with 10 mL dichloromethane according to our previous publications [55]. The ASL was determined as hydrolysate obtained after the hydrolysis of liquid samples with 4% H_2_SO_4_. The absorption of solubilized ASL was determined at a 205 nm wavelength using a spectrophotometer Lambda 25 (Perkin Elmer, Beaconsfield, UK) with 1 cm glass cells [56]. The acetic acid from hemicellulosic fraction was determined by dilution of liquid fraction with ultrapure water and then analysed by ion chromatography (IC, Metrohm 761 Compact, Methrom Ltd., Herisau, Switzerland)) with suppressed conductivity.

#### 3.6.6. Determination of Reducing Sugars from Enzymatic Hydrolysates

The liquid fraction resulted from the SSF process was subjected to a spectrophotometric analysis using a DNS (3,5-dinitrosalicylic acid) assay method for the reducing sugars determination. The concentration was determined by using Lambda 25 ultraviolet visible spectrometer (Perkin Elmer) according to the Miller method [57]. The DNS was prepared by mixing 40 mL water with 1 g of 3.5-dinitrisalycilic acid and 2.075 mL NaOH 50%. The obtained solution was mixed with 30 g potassium and sodium tartrate and the final volume of solution was 100 mL with water. 1 mL hydrolysate solution was mixed with 3 mL DNS reagent and the solution was boiled for 5 min. The solution absorbance was measured at 540 nm. The reduced sugars were calculated using Equation (1):

(1) $$\mathrm{Reduced}\;\mathrm{sugars}\;(\%)=\frac{W_{S}}{W_{1\;\times \;1000}}\;100$$

where: W_S_ is the quantity of sugars determined by reaction with DNS (mg), *W*_1_ is the initial sample (g). W_S_ is calculated with Equation (2):

W_S_ = C_DNS_ V (2)

where: C_DNS_ is concentration of sugars (mg mL^−1^), V is the total volume of hydrolysates (mL) and 0.9 is the conversion factor of the cellulose transformation into glucose.

#### 3.6.7. Determination of Ethanol Content

The liquid fraction obtained after the SSF process was analysed for ethanol by gas chromatography coupled with flame ionization detector (GC-FID) by using a 7890A gas chromatograph (Agilent Technologies, Palo Alto, CA, USA) equipped with a CTC Combi PAL autosampler (CTC Analytics AG, Zwingen, Switzerland) and FID detector. 10 µL of sample were introduced in a headspace sample vial and then they were completely evaporated by heating up to a temperature of 105 °C. The ethanol yield (%) was calculated by the Equation (3):

(3) $$Y_{\mathrm{Et}}\;=\frac{{\mathrm{Et}}_{f}-{\mathrm{Et}}_{0}}{0.51\;.f.\;B1.1.111}\;\times \;100$$

where Et_f_ is the ethanol concentration produced during the fermentation (g L^−1^), Et_0_ is the ethanol concentration at the beginning of the fermentation (g L^−1^), which is zero, *B* is the dry biomass concentration at the beginning of the fermentation (g L^−1^), *f* is the glucan fraction of dry biomass (g g^−1^), 0.51 is the conversion factor of the glucose to ethanol and 1.111 is stoichiometric coefficient of the glucan transformation to glucose.

### 3.7. Structural Characterization of Pretreated and Delignified Vine-Shoot Waste

#### 3.7.1. Scanning Electron Microscopy (SEM)

The vine-shoot wastes, before and after the pretreatment and delignification were examined using a scanning electron microscope (SEM VEGAS 3 SBU, Tescan, Brno-Kohoutovice, Czech Republic) with EDX detector. The samples were deposited on a double-sided conductive carbon tape on to aluminium stubs and analysed. All samples were dried at 105 °C before the analysis.

#### 3.7.2. X-ray Diffraction (XRD)

The X-ray diffraction (XRD) patterns were recorded using a D8 Advance diffractometer (Bruker, Karlsruhe, Germany), operating at 40 KV and 40 mA, with CuK_α_ radiation (λ = 1.5406 Å), at room temperature. The crystallinity index (CrI) was calculated according to Segal, using Equation (4) [58]:

(4) $$\mathrm{CrI}\;(\%)=\left(\frac{I_{002\;}-I_{am}}{I_{002}}\right)\;\times \;100$$

where *I*_002_ is the maximum intensity of the (002) diffraction peak (2θ ≈ 22.5°) and *I_am_* is the intensity scattered by the amorphous part of the sample (2θ ≈ 18.0°). The degree of crystallinity (χ_c_) was calculated as the relationship between the crystalline and amorphous region using Equation (5).

(5) $$\chi_{c}\;(\%)=\left(\frac{F_{c}}{F_{a\;}+F_{c}}\right)\;\times \;100$$

where χ_c_ is the degree of crystallinity, *F_c_* and *F_a_* are the area of the crystalline and amorphous regions [34]. The average crystallite size was calculated according to the Scherrer Equation (6):

(6) $$D_{c}=\frac{0.9\;\lambda }{\beta \;\cos\theta }$$

where D_c_ is the crystallite size, *λ* is the X-ray wavelength, *β* is the full width at half maximum intensity and θ is the Bragg angle.

### 3.8. Statistics

For the statistical processing of the data, the XLStat (Addinsoft, Paris, France), Microsoft Excel add-on software (BASIC+, 2019.3.2) was used. Principal component analysis with Varimax rotation and hierarchical clustering analysis were used to interpret the structure of the principal dataset.

## 4. Conclusions

Autohydrolysis pretreatments have been applied to shoot waste of eight vine plant varieties in order to obtain hemicellulosic sugars-containing liquors and spent solids as substrates for bioethanol production. Due to their high cellulose content, the vineyard cutting wastes can be used for the bioethanol production. The hemicellulosic sugars were characterized by the α and β anomers for each pentosic and hexosic sugar, HMF, furfural and ASL contents. The spent solids were delignified using sodium chlorite for lignin removal before the SSF process. The highest ethanol content was obtained at 37 °C. The untreated vine-shoot wastes were characterized by their contents of metals, elemental analysis, calorific value and ash, and the principal component analysis was applied for establishing a possible correlation between the vine plant varieties and their bioethanol production yields. All studied samples are rich in minerals (Na, K, Ca and Mg). In conclusion, the implementation of environmentally friendly methods to obtain a ready substrate for the bioethanol production will be the procedure of choice for the vine-shoot wastes valorisation.

## Figures and Tables

**Table 1:** Chemical compositions of vine-shoots waste and solid yield and chemical compositions of autohydrolysed vine-shoot waste obtained at 165 °C and 180 °C.

Temperature (°C)	*Sauvignon Blanc*	*Pinot Noir*	*Feteasca Regala*	*Busuioaca de Bohotin*	*Muscat Ottonel*	*Cabernet Sauvignon*	*Feteasca Neagra*	*Feteasca Alba*
Solid yield (% of raw material, dry biomass)								
165 °C	63.9 ± 7.6	62.6 ± 7.5	71.0 ± 8.0	70.2 ± 8.4	60.3 ± 6.6	65.3 ± 7.1	62.2 ± 6.7	66.2 ± 7.2
180 °C	57.3 ± 6.8	56.04 ± 6.7	64.3 ± 7.7	68.6 ± 7.9	52.3 ± 5.7	56.1 ± 6.2	57.6 ± 6.3	59.3 ± 6.5
Compositions of solid fraction resulted at 165 °C (% of autohydrolyzed biomass, dry biomass)								
Cellulose	53.0 ± 6.3	57.0 ± 5.8	55.3 ± 6.6	56.0 ± 5.9	49.3 ± 5.9	57.3 ± 6.8	51.3 ± 6.1	51.9 ± 6.2
Hemicelluloses	1.0 ± 0.1	1.0 ± 0.1	1.0 ± 0.1	1.5 ± 0.1	1.2 ± 0.1	1.3 ± 0.1	1.6 ± 0.1	1.0 ± 0.1
Lignin	45.0 ± 5.4	41.0 ± 4.0	43.2 ± 5.1	40.7 ± 4.8	36.3 ± 4.3	32.4 ± 3.8	41.3 ± 4.3	42.6 ± 5.1
Solid compositions	99.0 ± 9.3	99.0 ± 8.0	99.5 ± 8.4	98.1 ± 9.1	86.8 ± 9.6	91.0 ± 9.5	94.2 ± 7.8	95.5 ± 8.6
Compositions of solid fraction resulted at 180 °C (% of autohydrolyzed biomass, dry biomass)								
Cellulose	49.0 ± 5.8	53.0 ± 6.0	51.3 ± 5.4	52.0 ± 4.8	45.3 ± 5.4	53.3 ± 6.0	47.3 ± 5.6	47.9 ± 5.2
Hemicelluloses	-	-	0.1 ± 0.0	0.2 ± 0.01	-	-	0.1 ± 0.0	0.1 ± 0.0
Lignin	41.0 ± 4.9	37.0 ± 4.4	39.2 ± 4.5	36.7 ± 3.8	32.3 ± 3.8	28.4 ± 3.0	37.3 ± 4.3	38.6 ± 3.5
Solid compositions	90.0 ± 9.8	90.0 ± 8.6	90.6 ± 7.6	88.8 ± 8.7	77.6 ± 7.5	81.7 ± 9.8	84.7 ± 8.6	86.6 ± 7.6

Data represents mean ± standard deviation, *n* = 3.

**Table 2:** Mass balance for cellulose and lignin recovered in residual solids.

Vine-Shoot Variety	Cellulose Recovered after Pretreatment/Delignification (% of Vine-Shoot Waste, Dry Biomass)						Lignin Recovered after Pretreatment/Delignification (% of Vine-Shoot Waste, Dry Biomass)					
	150 °C		165 °C		180 °C		150 °C		165 °C		180 °C	
Sauvignon Blanc	23.2 ± 2.5	19.0 ± 1.6	33.5 ± 2.8	21.7 ± 2.3	25.3 ± 2.7	20.0 ± 1.8	17.3 ± 1.9	1.6 ± 0.2	28.5 ± 2.6	1.3 ± 0.1	21.2 ± 2.5	0.9 ± 0.1
Pinot Noir	26.6 ± 2.3	22.1 ± 2.4	35.3 ± 2.0	24.7 ± 2.5	26.7 ± 2.3	22.7 ± 2.31	18.0 ± 1.3	1.6 ± 0.1	25.4 ± 3.1	1.9 ± 0.1	18.7 ± 1.9	1.0 ± 0.1
Feteasca Regala	29.9 ± 3.0	18.9 ± 1.8	39.1 ± 3.2	24.7 ± 2.6	29.9 ± 3.0	21.3 ± 2.0	22.4 ± 2.1	1.6 ± 0.2	30.5 ± 2.8	1.4 ± 0.1	22.8 ± 2.6	0.9 ± 0.2
Busuioaca de Bohotin	25.4 ± 2.2	19.4 ± 2.0	38.6 ± 3.5	22.3 ± 1.8	31.7 ± 2.6	19.1 ± 2.7	17.3 ± 1.7	1.4 ± 0.1	28.0 ± 2.3	1.1 ± 0.1	22.4 ± 2.4	0.9 ± 0.1
Muscat Ottonel	16.1 ± 1.6	20.0 ± 1.8	25.8 ± 2.8	23.3 ± 1.6	28.4 ± 2.4	20.3 ± 2.4	11.1 ± 2.9	1.3 ± 0.2	19.0 ± 1.7	1.1 ± 0.1	13.1 ± 1.8	0.8 ± 0.2
Cabernet Sauvignion	22.9 ± 2.3	20.1 ± 1.8	34.0 ± 3.0	24.3 ± 2.6	24.4 ± 2.6	20.9 ± 1.7	11.1 ± 1.3	1.6 ± 0.1	19.3 ± 2.1	0.8 ± 0.3	13.0 ± 1.6	0.9 ± 0.1
Feteasca Neagra	21.1 ± 2.1	18.2 ± 1.5	30.1 ± 2.9	20.8 ± 2.4	23.1 ± 1.8	18.0 ± 2.3	16.4 ± 1.8	1.5 ± 0.1	24.2 ± 2.0	0.7 ± 0.4	18.2 ± 2.2	0.8 ± 0.2
Feteasca Alba	21.4 ± 2.5	18.1 ± 1.9	32.8 ± 3.1	20.0 ± 2.2	24.6 ± 2.1	18.2 ± 1.8	17.1 ± 2.0	1.1 ± 0.1	26.9 ± 2.4	0.6 ± 0.2	19.8 ± 2.3	0.9 ± 0.1

(Data represents mean ± standard deviation, *n* = 3).

**Table 3:** The degree of crystallinity (χ_c_), the crystallinity index (CrI) and crystallite size (D_C_) calculated from XRD data of untreated, delignified vine-shoot waste and pretreated at 150, 165 and 180 °C.

Sample	χ_c_ (%)	CrI (%)	Dc (nm)
(a) Untreated vine-shoot waste	44.5	78.6	3.71
(b) Autohydrolized and delignified vine-shoot waste (at 165 °C)	46.6	65.9	3.61
(c) Autohydrolized vine-shoot waste at 150 °C	45.0	71.3	4.52
(d) Autohydrolized vine-shoot waste at 165 °C	44.2	63.6	4.87
(e) Autohydrolized vine-shoot waste at 180 °C	41.0	61.0	5.24

**Table 4:** Bioethanol concentration obtained in this study and the results reported by literature.

Raw Material	Pretreatment Conditions	Concentration of Ethanol	Reference
Vine pruning residue (from Portugal)	Autohydrolysis I two stages: (a) 180 °C, 60 min 6:1 (solid:liquid ratio) and (b) 180–200 °C, 30–40 min-SSF process	13.1 kg ethanol 100 kg^−1^ vine punning	[30]
Vineyard pruning (from Italy)	Alkaline pretreatment (NaOH), enzymatic hydrolysis	202 g glucose kg^−1^ of raw material	[42]
Grape stalks (from *Bonarda* and *Barbera* red cultivars, Italy)	Autohydrolysis 121 °C, acid hydrolysis with 2% H_2_SO_4_ and fermentation with *Debaryomyces nepalensis* NCYC 1026	20.84 g L^−1^ ethanol (0.35 g g^−1^ monomeric sugars)	[43]
Hornbeam wood (*Ostrya carpinifolia*)	Steam explosion (230 °C, 28 bar),	25 L ton^−1^ dry material	[44]
Vine-shoot waste (from Romania)	Autohydrolysis (150, 165, 180 °C), delignification with sodium chlorite, SSF process	3.0–6.0 kg ethanol 100 kg^−1^ vine shoot waste	This study

**Table 5:** Metals (mg kg^−1^) content and calorific value (CV, MJ kg^−1^) of vine-shoot waste variety (data represents mean ± standard deviation, *n* = 3 parallel measurement).

Component	Sauvignon Blanc	Pinot Noir	Feteasca Regala	Busuioaca de Bohotin	Muscat Ottonel	Cabernet Sauvignon	Feteasca Neagra	Feteasca Alba
Na	252 ± 5.3	177 ± 6.2	363.8 ± 5.8	185 ± 4.3	191 ± 3.8	178 ± 6.0	200 ± 5.3	166 ± 4.2
Mg	840 ± 4.3	719.1 ± 5.2	1255 ± 6.0	630 ± 5.6	776 ± 6.5	865 ± 5.1	861 ± 4.2	696 ± 5.0
K	3414 ± 5.2	2282.6 ± 4.8	3241 ± 5.6	2209 ± 6.2	1788 ± 5.6	2359 ± 4.9	3210 ± 5.1	3356 ± 5.9
Ca	2862 ± 6.2	2011.5 ± 5.4	3450 ± 6.6	1782 ± 5.3	1622 ± 4.8	2668 ± 5.7	2159 ± 6.0	2018 ± 4.7
Al	67.0 ± 4.1	52.2 ± 3.2	60.7 ± 2.2	41.7 ± 4.0	56.8 ± 3.8	56.5 ± 3.4	53.5 ± 2.9	58.6 ± 3.7
CV	1.56 ± 0.08	1.3 ± 0.05	1.7 ± 0.04	1.44 ± 0.05	1.56 ± 0.09	1.57 ± 0.08	1.59 ± 0.06	1.6 ± 0.05

**Table 6:** Varimax rotated factor loadings of significant PCs.

Variable	PC1	PC2	PC3	PC4
Eigenvalue	7.96	3.77	2.94	2.47
Variability (%)	39.80	18.84	14.72	12.33
Cumulative (%)	39.80	58.65	73.36	85.69
Factor loadings after varimax rotation				
Ash	**0.925**	0.082	0.066	0.161
Extractable	−0.001	**−0.595**	**−0.570**	0.010
Lignin	−0.033	0.226	**0.918**	0.123
Cellulose	**0.508**	0.216	−0.083	−0.427
Hemicelluloses	−0.042	0.204	0.031	**0.898**
C	0.141	**−0.806**	−0.160	0.061
Protein	0.204	**0.833**	−0.206	0.463
CV	0.450	−0.052	0.224	0.345
Bioethanol	−0.233	**−0.614**	−0.199	−0.451
Fe	0.590	**0.762**	0.204	0.003
Cu	**0.665**	0.426	−0.259	0.486
Zn	0.174	**0.785**	0.344	−0.121
Na	0.516	0.244	**0.797**	0.057
Mg	**0.731**	−0.013	**0.607**	−0.021
K	0.429	0.130	0.168	**0.830**
Ca	**0.653**	0.209	**0.532**	0.295
Mn	**−0.951**	0.080	−0.036	−0.037
Al	**0.775**	0.455	−0.133	0.288
Sr	−0.079	0.003	**0.970**	0.023
Ba	0.132	−0.250	0.055	**0.790**
